# Reinke’s Oedema in Africa: A Scoping Review

**DOI:** 10.7759/cureus.78931

**Published:** 2025-02-13

**Authors:** Olorunleke M Arokoyo, Collins T Adeleye, Winifred U Hyacinth, Abdulahi Zubair, Abdulrahman Omodele, Frank Agada

**Affiliations:** 1 Otolaryngology, Surgery Interest Group Africa, Lagos, NGA; 2 Otolaryngology, York and Scarborough Teaching Hospitals NHS Foundation Trust, York, GBR; 3 Internal Medicine, Doncaster and Bassetlaw Teaching Hospitals NHS Trust, Doncaster, GBR; 4 Neurosurgery, Surgery Interest Group of Africa, Lagos, NGA; 5 Division of Otorhinolaryngology, Department of Surgery, Ahmadu Bello University/ Ahmadu Bello University Teaching Hospital, Zaria, NGA

**Keywords:** africa, ent, epidemiology, reinke's oedema, scoping review

## Abstract

Reinke’s oedema is a benign pathology of the vocal fold characterised by polypoidal degeneration of its subepithelial space, with resulting dysphonia and in severe cases dyspnoea. It is more common among females and smoking is a strong risk factor. Multiple conditions of the larynx ranging from benign to malignant diseases, which can also present with voice changes and dyspnoea, need to be ruled out when evaluating a patient for Reinke’s oedema. The quality of evaluation can be directly influenced by the level of training and experience of the assessor and the diagnostic resources available. This can pose a challenge for the evaluation and management of Reinke’s oedema in Africa, as there is a significantly low ENT surgeon-to-patient ratio within the continent, and a lot of health settings are resource-constrained. This review aimed to examine the available literature on the epidemiology, management, and outcomes of Reinke’s oedema in Africa.

A literature search on Reinke’s oedema was conducted on PubMed, Ovid Online, and African Journal Online, from inception till date. Articles were screened for their relevance to the epidemiology, management, and outcomes of Reinke’s oedema in Africa. Results were reported according to the Preferred Reporting Items for Systematic Reviews and Meta-Analyses (PRISMA) extension for scoping reviews. No article satisfied the eligibility criteria used in this review. The dearth of literature from this scoping review could be reflective of the significantly low reach and availability of ENT services in Africa, the low percentage of females who smoke in Africa when compared to other continents, and the relatively rare nature of Reinke's oedema. Our study has established a gap in the literature and highlights a need for further research.

## Introduction and background

Reinke’s oedema is a benign pathology of the vocal fold characterised by polypoidal degeneration of its subepithelial space, with resulting dysphonia and in severe cases dyspnoea [1]. The mechanism by which the disease develops is not fully understood [2]. However, it is known that smoking is the strongest risk factor, with laryngopharyngeal reflux and vocal cord abuse documented as associated factors [3]. It has a significantly higher female preponderance [2,3].

Voice change or difficulty with breathing, which are presenting complaints of Reinke’s oedema, can be caused by multiple differential diagnoses ranging from malignant conditions of the larynx to benign conditions like polyps and infections. Hence, proper evaluation is required to diagnose Reinke’s oedema confidently. The quality of evaluation will therefore be influenced by the level of training and experience of the assessor as well as diagnostic resources available. These factors also affect the availability, choice, quality, and outcome of care after a diagnosis has been reached. Treatment is aimed at improving dysphonia and includes surgical options, such as microdebridement, CO_2_ laser excision, cold steel excision, and intralesional injection [4]. Also, ensuring smoking cessation and providing speech therapy is key to improving outcomes [3-6]. It is therefore imperative that the expertise and resources be available to achieve good outcomes given the relative rarity of the disease, despite its benign nature. This poses a challenge for evaluating and managing Reinke’s oedema in Africa, as many health settings are resource-constrained, with expertise outstretched by workload or unavailable. This likely explains why readily citable data on Reinke’s oedema are from studies done outside the continent. This review aims to examine the available literature on the epidemiology, management, and outcomes of Reinke’s oedema in Africa.

## Review

Methods

This study was conducted using the Preferred Reporting Items for Systematic Reviews and Meta-Analyses extension for Scoping Reviews (PRISMA-ScR) checklist. A literature search was conducted with the following databases - PubMed, Ovid Online, and African Journal Online. Articles included in the search were limited to those published in English, regarding Reinke’s oedema from inception till date. 

This study included full-text journal articles on Reinke’s oedema. There were no restrictions on the age of patients included or the date of publication. Articles with the following characteristics were excluded as they were outside the scope of this review: published in any other language than English language; did not include patients from Africa or did not have disaggregated data about the African population; did not discuss or have any data on Reinke’s oedema; did not discuss the epidemiology, management, or outcomes of patients with Reinke’s oedema; and studies not conducted in Africa. 

The following article types were also excluded: abstracts, conference presentations, case reports, commentaries, letters to editors, reviews, and meta-analyses. The search strategy was jointly devised by the authors and is summarized below (Table 1). Two reviewers working in pairs screened titles and abstracts to limit bias, with any lack of consensus discussed with a third reviewer. The final search results were exported to Rayyan.ai (a systematic review website) for the removal of duplicates and screening of articles. Data on the prevalence, management, and outcomes were extracted into an Excel data sheet. No article satisfied the eligibility criteria used in this review. 

**Table 1:** Strategy of research

Keywords	Database	Results
Reinke’s oedema	PubMed	375
Reinke’s oedema	Ovid online (all resources)	204
Reinke’s oedema	African Journal Online	1

The alternate keyword search with (Reinke’s oedema) AND (Africa) did not yield any results on the databases either.

Results

**Figure 1:**
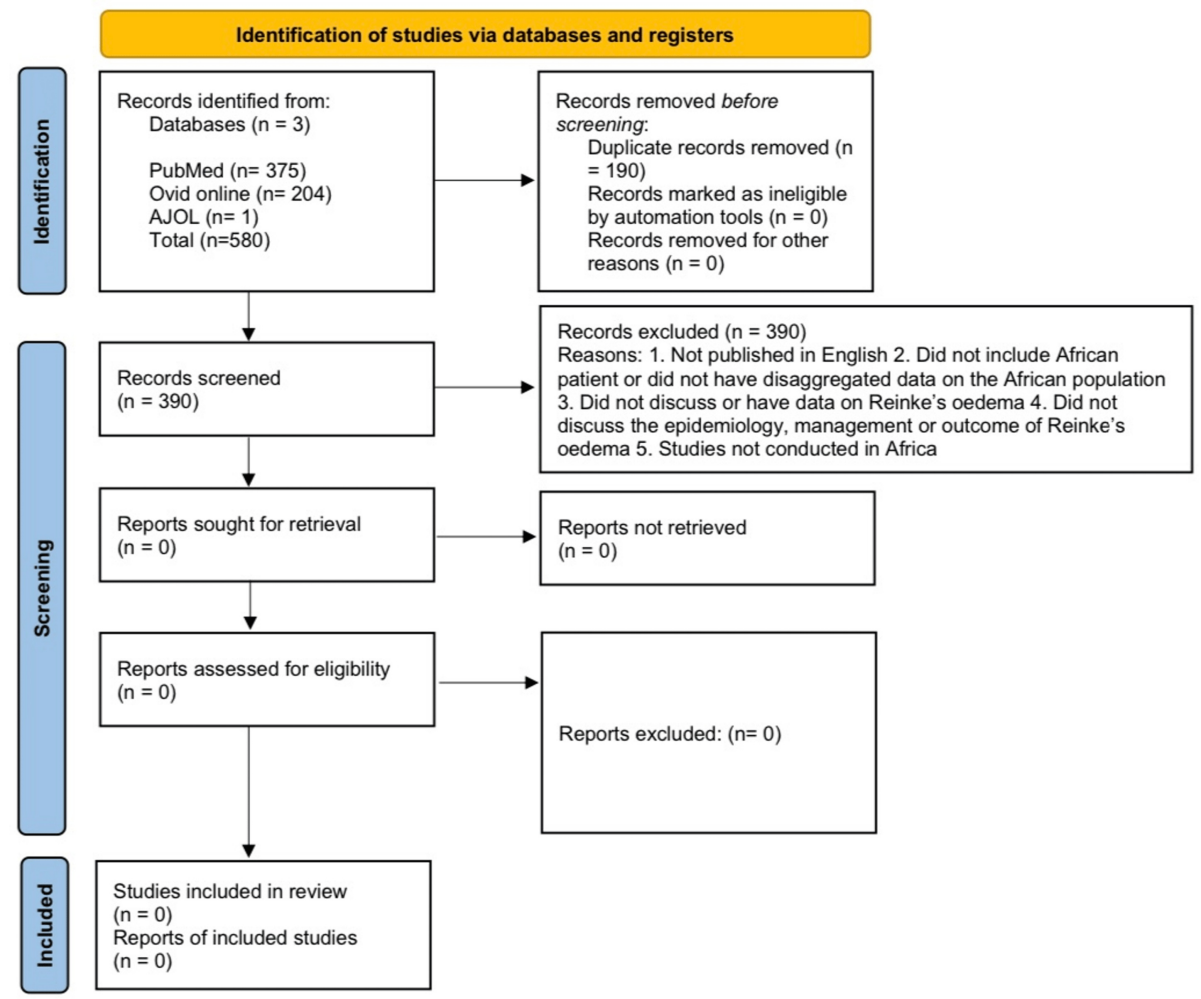
PRISMA 2020 flow diagram for new systematic reviews incorporating database and register searches only PRISMA: Preferred Reporting Items for Systematic Reviews and Meta-Analyses; AJOL: African Journal Online.

The search of databases yielded a total of 580 articles, and 190 duplicates were removed. After screening abstracts, no study made it to full-text screening as none satisfied the eligibility criteria for inclusion (Figure 1). Our findings reveal the dearth of literature regarding the epidemiology, management, and outcomes of Reinke’s oedema in Africa.

Discussion

The relatively low number of studies on the condition could be due to the rarity of Reinke’s oedema [7]. Reinke’s oedema has been strongly associated with smoking and it is much more common among females. Another factor that could be contributing to the low number of studies on this condition in Africa is the low prevalence of smoking among females in Africa (1.4%) compared to America (10.2%) and Europe (17.5%) [8]. The level of expertise and equipment required to successfully make a diagnosis after ruling out other conditions which could present similarly could also be contributing factors to the relatively low number of studies. This is particularly true on the African continent, as it is estimated that the majority of patients do not have access to much-needed ENT services, with training opportunities being very limited, in addition to very heavy demand on the available workforce (1.2 million patients per ENT surgeon in Sub Saharan Africa) [9-13]. Also, little progress has been made in mitigating these problems, despite the estimated population growth to 1.68 billion by 2030, from its 2015 estimate of 1.19 billion [11].

The dearth of literature established by this scoping review on Reinke’s oedema can be reflective of multiple factors, including the low level of ENT service available and the nature of health systems across the continent. Increased and prudent investment in equipment and workforce for ENT services, with increased reporting of ENT diseases encountered and treatment given, can help improve the current picture [12].

Limitations

Although this review was carried out through searches conducted on three databases, which put together, represent a robust resource, utilising additional databases could increase results.

## Conclusions

This scoping review on Reinke’s oedema in Africa has established a dearth of literature, with a need for further research on its epidemiology, management, and outcomes through various study designs such as case-control studies and cohort studies.
